# LeMYC2 acts as a negative regulator of blue light mediated photomorphogenic growth, and promotes the growth of adult tomato plants

**DOI:** 10.1186/1471-2229-14-38

**Published:** 2014-01-31

**Authors:** Nisha Gupta, V Babu Rajendra Prasad, Sudip Chattopadhyay

**Affiliations:** 1 National Institute of Plant Genome Research, New Delhi 110067, India; 2 Department of Biotechnology, National Institute of Technology, Mahatma Gandhi Avenue, Durgapur 713209, India

## Abstract

**Background:**

Arabidopsis ZBF1/MYC2bHLH transcription factor is a repressor of photomorphogenesis, and acts as a point of cross talk in light, abscisic acid (ABA) and jasmonic acid (JA) signaling pathways. MYC2 also functions as a positive regulator of lateral root development and flowering time under long day conditions. However, the function of MYC2 in growth and development remains unknown in crop plants.

**Results:**

Here, we report the functional analyses of LeMYC2 in tomato (*Lycopersicon esculentum*). The amino acid sequence of LeMYC2 showed extensive homology with Arabidopsis MYC2, containing the conserved bHLH domain. To study the function of LeMYC2 in tomato, overexpression and RNA interference (RNAi) *LeMYC2* tomato transgenic plants were generated. Examination of seedling morphology, physiological responses and light regulated gene expression has revealed that LeMYC2 works as a negative regulator of blue light mediated photomorphogenesis. Furthermore, LeMYC2 specifically binds to the G-box of *LeRBCS-3A* promoter. Overexpression of LeMYC2 has led to increased root length with more number of lateral roots. The tomato plants overexpressing LeMYC2 have reduced internode distance with more branches, and display the opposite morphology to RNAi transgenic lines. Furthermore, this study shows that LeMYC2 promotes ABA and JA responsiveness.

**Conclusions:**

Collectively, this study highlights that working in light, ABA and JA signaling pathways LeMYC2 works as an important regulator for growth and development in tomato plants.

## Background

Plant growth and development are adaptive to changes in ambient light conditions. Light is the energy source of photosynthesis, and is also an important environmental factor for plant growth and development [1-6]. Plants can respond to various light parameters including intensity, direction, duration and spectral quality, and modulate the developmental processes accordingly. The light signals are perceived by at least four distinct families of photoreceptors including red (R)/far-red (FR) light-sensing phytochromes, UV-A/blue light-absorbing cryptochromes, phototropins, and UV-B light absorbing UVR8 in Arabidopsis [4,7-9].

The economic importance of tomato makes it an attractive target for crop improvement by increasing the disease resistance, nutritional content and productivity by genetic manipulation [10-13]. Studies on increase in fruit nutritional value have mostly been carried out through modulation of the expression of structural or regulatory genes of specific pathway [14-21]. The tomato mutants such as *hp1* and *hp2* with hypersensitivity to light and elevated pigmentation have been reported [22]. It was subsequently shown that HP1 and HP2 are homologous to DDBI and DET1, respectively [23-27]. Reduced expression of *HP1/DDB1* by RNAi strategy has been shown to enhance pigmentation in tomato fruits [27]. It has been demonstrated further that both HP1/LeDDB1 and HP2/LeDET1 are essential components of a tomato CUL4-based E3 ligase complex, where LeDDB1 is associated with tomato CUL4 and DET1 [27].

The light signaling components including photoreceptors and central regulators have been shown to have strong potential in crop improvement [28]. Overexpression of *Arabidopsis* PhyB photoreceptor in potato was shown to increase the yield, both in gross weight and in number of tubers [29]. Four cryptochrome genes have been identified in tomato: two *CRY1* like (*CRY1a* and *CRY2b*), one *CRY2* and one *CRY-DASH* gene [30-32]. Role of CRY1 includes its importance in seedling photomorphogenesis, anthocyanin accumulation and plant development, however it does not show any effect on flowering time or fruit pigmentation [33]. Reduced level of cry1 in brassica transgenic plants led to increased plant height and lower level accumulation of anthocyanin [34]. Tomato CRY2 possesses similar but distinct functions in Arabidopsis. Overexpression of CRY2 in tomato transgenic plants display short hypocotyl and reduced internode distance, overproduction of anthocyanin and chlorophyll in leaves, and of flavonoids and lycopene in fruits [25,27,33,35-37]. It also shows strong effect on the expression of stress related gene products in response to diurnal cues [38]. Tomato *CRY-DASH* is expressed at early stages of tomato development and contributes significantly in the control of circadian machinery with a light regulated transcription [32]. Two light signaling components in tomato, LeCOP1LIKE and LeHY5, which antagonistically regulate the fruit pigmentation, have been identified [24]. RNAi-mediated down-regulation of *DET1* and *LeCOP1LIKE* resulted in increased carotenoid levels in tomato fruits [24,26].

Arabidopsis MYC2 is a bHLH transcription factor that works downstream to cry1 and cry2 photoreceptors [39]. MYC2 acts as a point of crosstalk among multiple signaling pathways such as light, ABA, JA and Ethylene-Jasmonate [39-43]. Although the function of MYC2 has been mainly investigated in Arabidopsis, *AtMYC2* orthologs/homologs that have been characterized from other monocots or dicot plants have recently been reported. The recent reports from dicots have implied a broadly conserved role of MYC2 with context to its function in JA signaling pathways [43-46]. *TcJAMYC*, the homolog of *AtMYC2*, is a key candidate gene to increase paclitaxel (anticancer drug) accumulation in *Taxus*cell cultures [47]. Three AtMYC2 orthologs, NbbHLH1 and NbbHLH2 from *Nicotiana benthamiana* and NtMYC2 from *Nicotiana tabacum,* have been shown to regulate the expression of nicotine biosynthesis genes in the roots [48]. CrMYC2, a MYC2 ortholog from *Catharanthus roseus,* regulates the JA responsiveness of genes involved in the regulation of alkaloid biosynthesis [45]. Two MYC2 orthologs, MaMYC2a and MaMYC2b, in the regulation of JA-induced chilling tolerance in banana (*Musa acuminate*) fruit have been reported [49]. A recent study in maize (*Zea mays*) implicated MYC7, a putative MYC2 ortholog, in systemic signaling activation in response to insect elicitors [50]. However, the functional role of MYC2 in growth and development is practically unknown in crop plants [51]. Here, we have carried out the functional analysis of LeMYC2 using transgenic tomato plants. Our results suggest that LeMYC2 acts as a negative regulator of blue light–mediated photomorphogenic growth.

## Results

### LeMYC2 acts as a negative regulator of blue light mediated seedling development

Arabidopsis ZBF1/MYC2 has been shown to work as a repressor of cryptochrome-mediated blue light (BL) signaling [39]. To determine the function of MYC2 in growth and development in crop plants, we cloned *LeMYC2/ZBF1*from tomato (*Lycopersicon esculentum* cv: Pusa Ruby; Indian Agricultural Research Institute, New Delhi) by reverse transcriptase-PCR. The coding sequence of *LeMYC2* cDNA appeared to be a full-length cDNA (GeneBank Accession number KF428776) encoding a protein of 689 amino acids (predicted molecular mass of 75.7 kD) with a bHLH domain. The amino acid sequence of LeMYC2 has 93%, 66% and 53% identity to JAMYC2 *(Solanum tuberosum;* CAF 74710), CrMYC2 (*Catharanthus roseus*; AAQ 14332) and AtMYC2 (*Arabidopsis thaliana;* At1g 32640), respectively [40,44,45] (Additional file 1: Figure S1).

**Figure 1:**
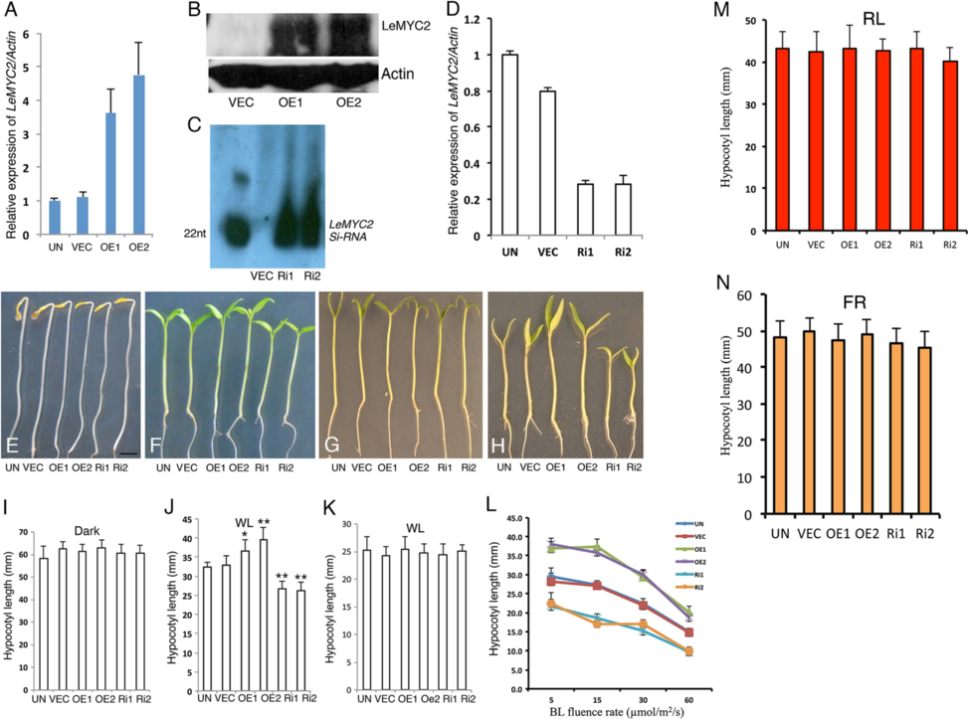
**LeMYC2 mediated regulation of hypocotyl elongation during early tomato seedling development. A**, Real time PCR analysis for *LeMYC2* transcript levels in 6-day-old seedlings of various LeMYC2 overexpresser transgenic lines grown in BL. *LeActin* used as control. Error bars represent SD. Number of independent experiments with similar results is (n ≥ 3). **B**, Western blot analysis of control (VEC) and LeMYC2 overexpresser transgenic lines (OE1 and OE2). The gel blot was probed with anti-MYC2 antibodies. Anti-ACTIN antibody was used to probe ACTIN immunoblot. **C**, Detection of gene-specific 22 nucleotide siRNAs by Northern blot analysis in RNAi transgenic plants targeting *LeMYC2. LeMYC2* was used as probe for the detection of siRNAs. **D**, Real time PCR analysis for *LeMYC2* transcript levels in 6-day-old seedlings of various LeMYC2 RNAitransgenic lines grown in BL. For experimental detail, see legend to **A**. **E** to **H**, Visible phenotypes of 6-day-old seedlings grown in darkness, white light (WL: 5 μmol/m^2^/s), white light (WL: 30 μmol/m^2^/s) and blue light (BL: 30 μmol/m^2^/s), respectively. In each panel, from left to right seedlings of UN (Untransformed), VEC (vector control), OE1 (LeMYC2OE1), OE2 (LeMYC2OE2),Ri1 (LeMYC2RNAi1) and Ri2 (LeMYC2RNAi2) lines are shown. Scale bar, 1 cm. **I** to **L**, Quantification of hypocotyl length of 6-day-old seedlings as shown in **A** to **D**, respectively. About 15 seedlings of each line were used for the measurement of hypocotyl length. Error bars indicate standard deviation (SD, n = 6). Asterisks in Figure F indicate that OE1, OE2, Ri1 and Ri2 are significantly different from the vector control (*P < 0.05 and **P <0.01, Student’s t test). The experiment was repeated for 4 times. **M** and **N**, Quantification of hypocotyl length of 6-day-old seedlings grown in RL (30 μmol/m^2^/s) and FR (20 μmol/m^2^/s), respectively. For experimental detail, see legend **E** to **H**.

To determine the physiological function of LeMYC2 in light-controlled seedling development, 22 tomato (*Lycopersicon esculentum)* transgenic lines overexpressing *LeMYC2*, 10 transgenic lines under-expressing *LeMYC2* (RNAi lines) and 8 vector control transgenic plants were generated. These independent lines were allowed to self-fertilize and the T2 seeds were collected for further studies. Since Arabidopsis *atmyc2* null mutants display relatively weak phenotype in photomorphogenic growth [39,52], two overexpresser lines (OE1 & OE2) with highest level of LeMYC2, and two RNAi lines (Ri1 & Ri2) with maximum down-regulation of *LeMYC2* transcript were selected in this study for further analysis. Analyses of *LeMYC2* transcript and protein levels in OE1 and OE2 transgenic lines showed higher level of transcript and protein accumulation as compared to control transgenic plants (Figure 1A and B). The RNAi transgenic lines including Ri1 and Ri2 showed reduced accumulation of *LeMYC2* transcript and higher-level accumulation of *LeMYC2 SiRNA* (Figure 1C-D).

To determine the possible role of LeMYC2 in tomato seedling development, we examined the morphology of 6-day-old LeMYC2 overexpresser and RNAi transgenic seedlings grown in dark, white light (WL) and at specific wavelengths of light. As shown in Figure 1E and I, no morphological difference was observed between control and LeMYC2 transgenic seedlings in the darkness. However, LeMYC2 transgenic seedlings displayed altered hypocotyl length in WL irradiation. Whereas the overexpresser transgenic seedlings showed reduced sensitivity, the RNAi transgenic seedlings displayed hypersensitive response with shorter hypocotyl especially at lower fluences (5 μmol/m^2^/s) of WL (Figure 1F and J). This altered hypocotyl length however was not observed at higher fluences (30 μmol/m^2^/s) of WL (Figure 1G and K). Whereas enhanced inhibition in hypocotyl elongation of RNAi seedlings was observed in BL, the overexpresser transgenic seedlings displayed elongated hypocotyl as compared to control seedlings (Figure 1H and L). While examined the hypocotyl length in red (RL) or far red light (FR) grown seedlings, no significant difference was observed between transgenic and control seedlings (Figure 1M and N). Taken together these results suggest that LeMYC2 acts as a negative regulator of BL-mediated inhibition of hypocotyl elongation. These results further indicate that although MYC2 does not play the negative regulatory role in WL-mediated inhibition of hypocotyl elongation in Arabidopsis [39], LeMYC2 works as a negative regulator of WL-mediated inhibition of hypocotyl elongation at lower fluences of WL.

### Down regulation of *LeMYC2* leads to higher level of chlorophyll and anthocyanin accumulation, and light regulated gene expression

**Figure 2:**
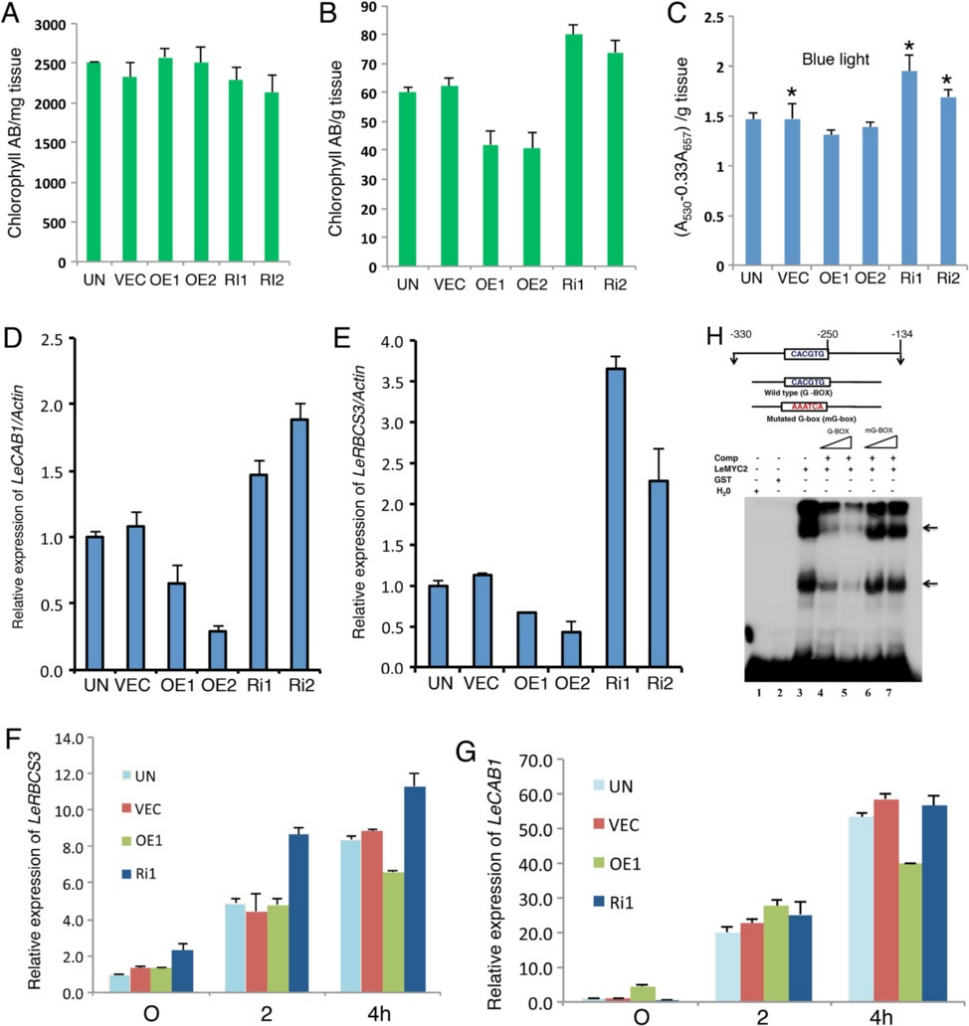
**Physiological characterization of LeMYC2 transgenic lines. A** and **B**, Accumulation of chlorophyll in 6-day-old seedlings grown in white light (WL: 30 μmol/m^2^/s) and blue light (BL: 30 μmol/m^2^/s), respectively. **C**, Accumulation of anthocyanin in 6-day-old seedlings grown in BL (30 μmol/m^2^/s). Asterisks indicate that Ri1 and Ri2 are significantly different from the vector control (*P < 0.05, Student’s t test; *n* = 5). **D** and **E**, Real time PCR analysis for *LeCAB1* and *L*e*RBCS3* transcript levels in 6-day-old seedlings of various LeMYC2 transgenic lines grown in BL (30 μmol/m^2^/s). For experimental detail, see Figure 1A. **F** and **G**, The abundance of *LeRBCS3* and *LeCAB1* transcripts, *respectively,* from different transgenic seedlings grown in darkness for 5 days and then transferred to BL (30 μmol/m^2^/s) for 2 h and 4 h was determined by quantitative real time PCR. For experimental detail, see Figure 1A. **H**, LeMYC2 interacts with G-box containing *LeRBCS3A promoter*. Upper panel, Diagrammatic representation of 127 bp long *LeRBCS-3A promoter* fragment containing G-box used in the electrophoretic mobility shift assays. Lower panel, Gel shift assays using the GST-LeMYC2 and 127 bp G-box containing *LeRBCS-3A promoter* as probe. No protein was added in lane 1, and approximately 500 ng of GST protein was added in lane 2. In lanes (3–7) about 350 ng of GST-LeMYC2 protein was added. Competition was performed with 50 (lane numbers 4 and 6) and 100 (lane numbers 5 and 7) molar excess of wild type or mutated versions of 80 bp DNA fragment of *LeRBCS-3A* promoter as shown by the triangles in the figure. The arrowheads indicate the DNA-protein complex formed. The plus (+) and minus (−) signs indicate the presence and absence, respectively.

Two important physiological responses, accumulation of anthocyanin and chlorophyll, are regulated by light signaling pathways. To determine the possible role of *LeMYC2* on chlorophyll and anthocyanin accumulation, we measured the level of chlorophyll and anthocyanin in overexpresser and RNAi transgenic tomato seedlings. As shown in Figure 2A, the level of chlorophyll remained similar in control and transgenic seedlings in WL. However, the chlorophyll accumulation was strongly reduced in overexpresser transgenic lines, and showed elevated level of accumulation in RNAi lines in BL (Figure 2B). On the other hand, although no significant difference was observed in the accumulation of anthocyanin in overexpresser transgenic lines in BL, the level of accumulation was significantly higher in RNAi transgenic lines as compared to vector control plants (Figure 2C). Taken together, these results indicate that LeMYC2 acts as a negative regulator of chlorophyll and anthocyanin accumulation in BL.

Since LeMYC2 acts as a negative regulator of photomorphogenic growth in BL, we ask whether the expression of light inducible genes such as *CAB* and *RBCS* are modulated by bHLH transcription factor, LeMYC2. To test this, we performed quantitative real time PCR of 6-day-old seedlings grown in BL and steady state mRNA level of light inducible genes was estimated. The expression of *LeCAB1* and *LeRBCS-3A* was increased in LeMYC2 RNAi transgenic lines, and the level of expression was compromised in overexpresser transgenic seedlings as compared to control plants in BL (Figure 2D and E).

To further examine the BL-mediated induction of *LeCAB1*and *LeRBC-3A* expression, 5-day-old transgenic seedlings grown in darkness were transferred to BL for 2 h and 4 h, and the transcript levels were measured. The level of induction of *LeCAB1* and *LeRBCS-3A* was elevated in RNAi transgenic lines as compared to wild-type and overexpresser seedlings at various time points. Whereas about 6-fold induction in *RBCS-3A* expression was found in RNAi lines at 4 h, less than 4-fold and 3-fold induction was detected in the wild type and overexpresser backgrounds, respectively (Figure 2F). In the case of *LeCAB1*, the differential expression between the overexpresser and RNAi transgenic lines was not observed after exposure to BL for 2 h (Figure 2G). However, an approximately 60-fold induction was detected in both wild type and RNAi lines, whereas the level of induction was reduced to less than 40-fold in the overexpresser transgenic background at 4 h (Figure 2G). Taken together, these results suggest that *LeMYC2* plays a negative regulatory role in the BL-mediated expression of *LeCAB1* and *LeRBCS-3A*.

### LeMYC2 specifically binds to the G-Box of *LeRBCS-3A* promoter

To determine whether LeMYC2 is able to work as a transcription factor, we investigated the interaction of LeMYC2 with the G-box present in *LeRBCS-3A* minimal promoter. We used purified glutathione S-transferase-LeMYC2 (GST-LeMYC2) fusion protein and 147 bp promoter fragment containing the G-box of *LeRBCS3A* for gel shift assays. As shown in Figure 2H, whereas GST alone did not show any binding activity (lane 2), strong low mobility DNA-protein complexes were formed with GST-LeMYC2 fusion protein (lanes 3). This DNA-protein complexes were efficiently competed out by 50 and 100 molar excess of unlabelled G-box (lanes 4 and 5) but not with 50 and 100 molar excess of mutated G-box, mG (lanes 6 and 7). Taken together, these results suggest that LeMYC2 specifically interacts with the G-box of *LeRBCS-3A* minimal promoter.

### LeMYC2 positively regulates the root growth in tomato plants

**Figure 3:**
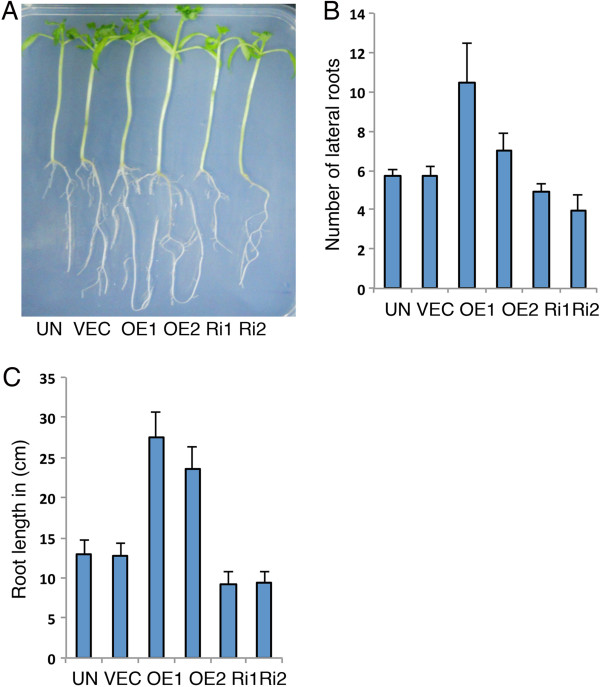
**Role of LeMYC2 in the regulation of root growth. A**, Visible phenotypes of root growth of 14-day-old control and various LeMYC2 transgenic plants grown in WL (100 μmol/m^2^/s). **B**, Quantification of number of lateral roots of LeMYC2 transgenic plants as shown in **A**. **C**, Quantification of root lengthof LeMYC2 transgenic plants as shown in **A**.

Arabidopsis *atmyc2* mutant plants develop significantly less number of lateral roots and exhibit short stature as compared to corresponding wild-type background [39]. We ask whether altered level of LeMYC2 leads to altered root morphology in tomato transgenic plants. Examination of root growth of 14-days old LeMYC2 RNAi transgenic plants revealed that the transgenic lines developed less number of lateral roots as compared to vector control plants (Figure 3A and B). Higher level of LeMYC2 protein in overexpresser transgenic plants led to formation of more lateral roots in comparison to control plants (Figure 3A and B). Although Arabidopsis *atmyc2* mutants do not show any altered root length, the LeMYC2 overexpresser transgenic plants showed drastically longer root, and the root length was significantly compromised in RNAi transgenic lines as compared to corresponding vector control plants (Figure 3A and C). Collectively, these results suggest that LeMYC2 positively regulates the lateral root formation and root length.

### Overexpression of LeMYC2 leads to more branches with reduced internode distance

**Figure 4:**
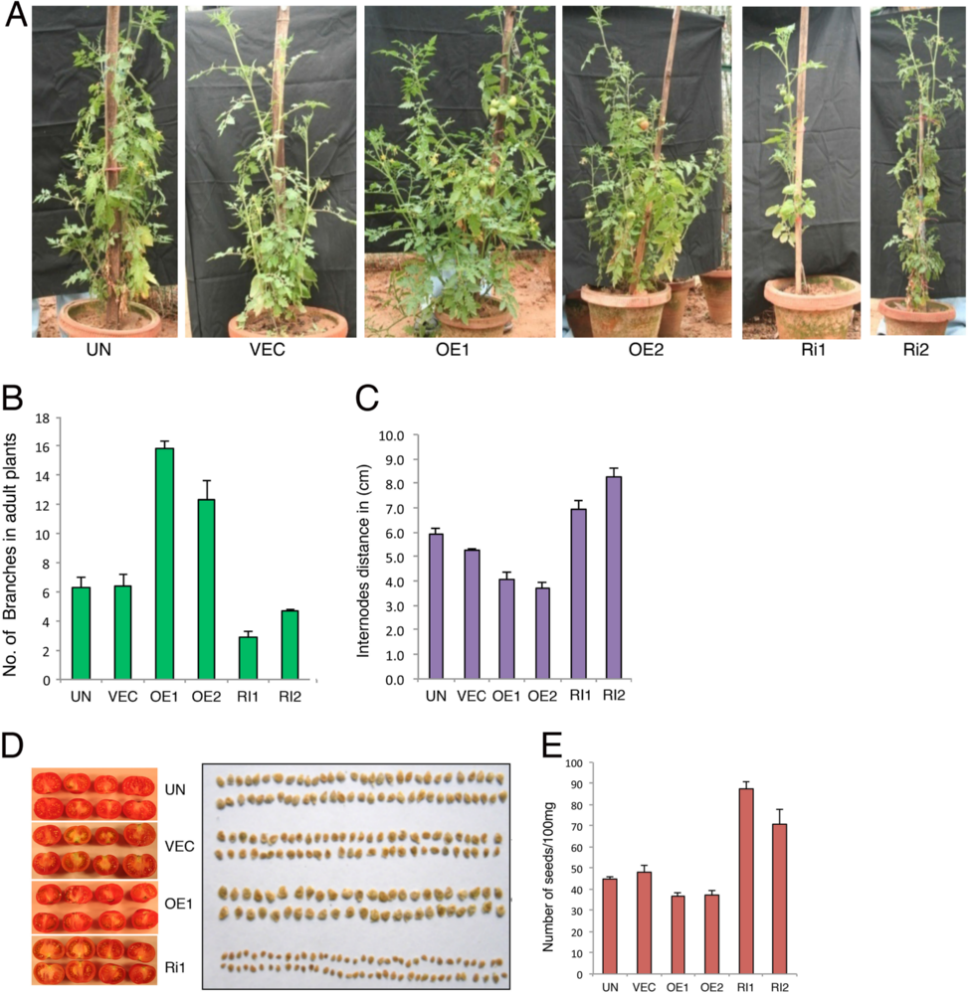
**Overexpression of LeMYC2 results in more branches. A**, Visible phenotypes of various LeMYC2 transgenic plants (T2 generation) at the adult stage. From left to right, plants of UN (Untransformed), VEC (vector control), LeMYC2OE1*,* LeMYC2OE2, LeMYC2Ri1 and LeMYC2Ri2 lines are shown. **B** and **C**, Quantification of number of branches **(B)** and inter-nodal length **(C)**, respectively. Error bars indicate SE (n = 6). **D**, Pictures of tomato fruits (left panel) and seeds (right panel) of control (UN and VEC) and LeMYC2 transgenic lines. **E**, Quantification of number of seeds per 100 mg in control and transgenic plants of LeMYC2. Error bars indicate SD (*n* = 5).

In Arabidopsis, *atmyc2* mutant adult plants display short stature as compared to wild-type control plants [39]. To investigate the role of LeMYC2 at the adult stage, phenotype of about three months old Green House plants was examined. No significant change in height was detected in RNAi or overexpression tomato transgenic plants as compared to control plants (Figure 4A). LeMYC2 RNAi transgenic plants however showed less number of branches, while overexpresser lines were branched with about twice as many branches as vector control plants (Figure 4A and B). The internode distance of the overexpresser transgenic plants was significantly reduced as compared to control plants, whereas it was drastically increased in the RNAi transgenic plants (Figure 4C). These results, taken together, suggest that LeMYC2 promotes formation of branches in tomato plants.

To determine whether the number of seeds per tomato fruit was altered in transgenic plants, we examined the number of seeds in ripe fruits from various transgenic and control plants. Although overexpresser or RNAi transgenic plants produced viable seeds, almost all of the seeds in the fruits of RNAi transgenic lines were smaller in size than the control plants. On the contrary, the size of the seeds in overexpresser transgenic lines was larger than the control plants (Figure 4D and E).

### Altered level of *LeMYC2* expression modulates ABA and JA responsiveness

**Figure 5:**
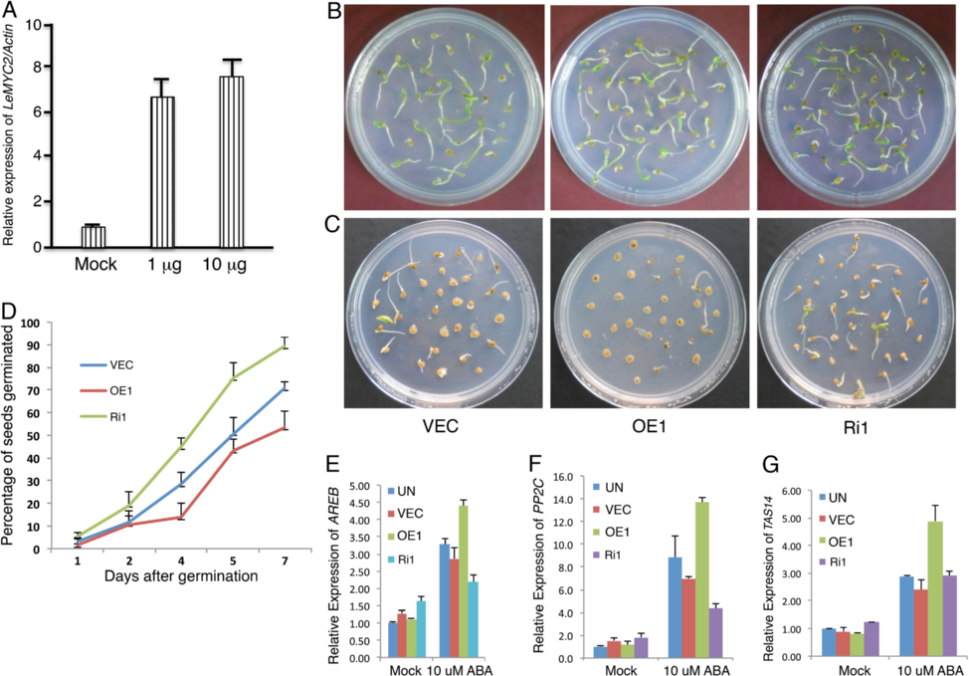
**ABA responsiveness of LeMYC2 transgenic plants. A**, Real time PCR analysis of *LeMYC2* expression in 6-day-old tomato seedlings treated with ABA for 48 h. No hormone treatment was used as control (Mock), and different concentrations of ABA are indicated. **B** and **C**, Photographs showing difference in the germination of 3-day-old control (VEC) and various LeMYC2 transgenic lines, grown in absence and presence of 5 μM ABA respectively. In both Figures, from left to right, seedlings of VEC (vector control), LeMYC2OE1 and LeMYC2Ri1 lines are shown. The seeds are considered to be germinated while the radicle tip had fully expanded the seed coat. The experiments were repeated thrice and similar results were obtained. The data shown is the representative of one of those experiments. **D**, Quantification of percentage of seeds germinated over a period of time as shown in **B**. The error bars indicate SD (*n* = 3). **E** to **G**, Real time PCR analysis of ABA marker genes, *AREB*(D), *PP2C*(E) and *TAS14*(F) from 6-day-old controls (UN and VEC) and various LeMYC2 transgenics in WL (60 μmol m^-2^ s^-1^) and transferred to MS or MS with 5 μM ABA for 6 h in WL. The error bars indicate SD (*n = 3),* normalized with *LeACTIN*. Accession numbers of genes are *AREB* (AY530758.1), *TAS14* (X51904.1) and *PP2C* (AI772677.1).

Arabidopsis MYC2 acts as a point of crosstalk in multiple signaling pathways including light, abscisic acid (ABA) and jasmonic acid (JA) [39-43]. To determine ABA mediated induction of *LeMYC2* expression, 6-day-old tomato seedlings were treated with ABA and the level of *LeMYC2* transcript level was monitored. The expression of *LeMYC2* was induced by ABA and the level of induction increased with higher concentration of ABA treatment (Figure 5A). To determine whether down-regulation of *LeMYC2* is able to alter the ABA responsiveness in transgenic tomato plants, freshly harvested seeds of overexpresser and RNAi transgenic lines were plated on MS plates without or with ABA. In the absence of ABA, the rate of germination of seeds in various lines was found to be similar (Figure 5B). However, 5 *μ*M of ABA reduced the rate of seed germination of control plants, and the effect was more severe in overexpresser transgenic lines (Figure 5C and D). On the other hand, down-regulation of *LeMYC2* in RNAi lines led to significantly less sensitivity to ABA with higher rate of seed germination (Figure 5C and D). These results indicate that LeMYC2 positively regulates ABA-mediated inhibition of seed germination. We then monitored the expression of ABA responsive genes in LeMYC2 transgenic lines. The expression of *AREB, PP2C* and *TAS14*[53-56] was significantly reduced in LeMYC2 RNAi transgenic lines as compared to control plants in the presence of ABA. Furthermore, the level of expression of these genes was significantly increased in LeMYC2 overexpresser transgenic background (Figure 5E-G). These results suggest that *LeMYC2* positively regulates the expression of ABA responsive genes such as *AREB, PP2C* and *TAS14.*

**Figure 6:**
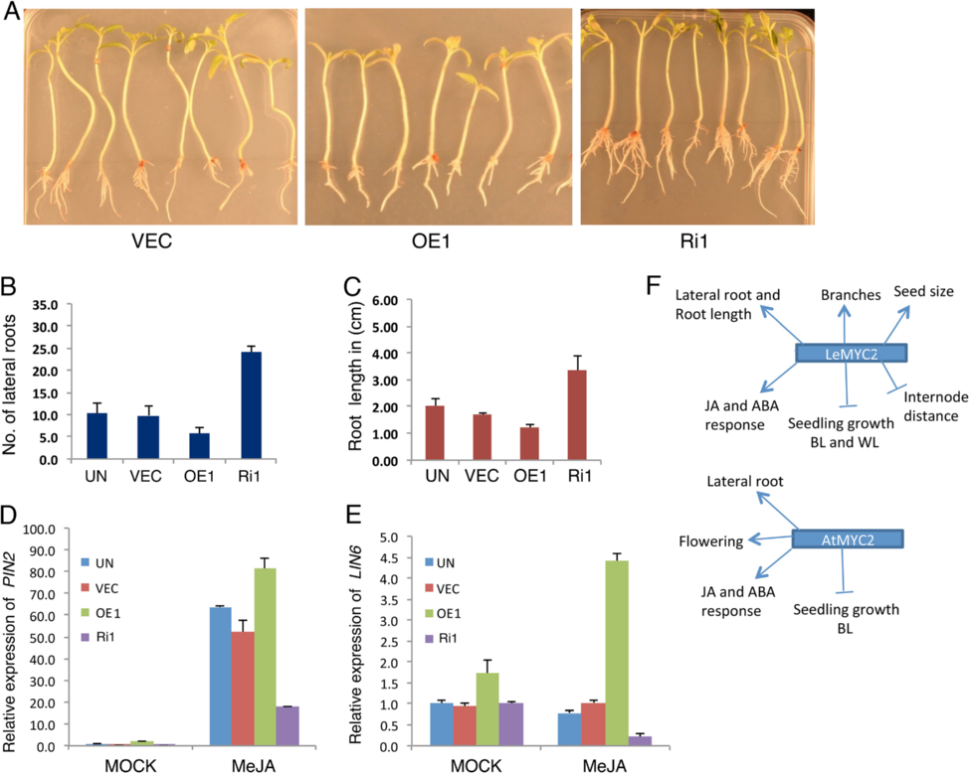
**JA responsiveness of LeMYC2 transgenic plants. A**, The root phenotype of 14-day-old constant WL (90 μmol/m^2^/s) grown various transgenic lines of tomato with control (VEC) grown in presence of 10 μM JA. From left to right, plants of VEC (vector control), LeMYC2OE and LeMYC2Ri1 lines are shown. **B** and **C**, Quantification of number of lateral roots **(B)** and root length **(C)**, respectively, of those shown in **A**. The experiments were repeated thrice and similar results were obtained, the data shown is the representative of one of those experiments. The error bars indicate SD (n = 15). **D** and **E**, Real-time PCR analysis of JA marker genes, *LIN6***(D)** and *PIN2***(E)** from 6-day-old controls (UN and VEC) and various LeMYC2 transgenic lines in WL (60 μmol/m^2^/s) and transferred to MS or MS with 50 μM JA for 6 h in WL. The error bars indicate SD (*n = 3),* normalized with *LeACTIN.* Accession numbers of genes are *LIN6 (AF566005)* and *PIN2* (*BG629234*)*.***F**, The model shows the function of MYC2 in tomato (LeMYC2) and Arabidopsis (AtMYC2).

Among plethora of functions of JA in plant growth and development, one is the inhibition of root growth. To investigate the JA responsiveness of LeMYC2 transgenic plants, we grew the plants in the presence of JA and monitored the root growth. JA caused root growth retardation with reduced length and number of lateral roots formed in control and overexpresser transgenic plants (Figure 6A-C). The effect was more severe in the overexpresser transgenic lines than the vector control plants (Figure 6A-C). On the other hand, the effect of JA-mediated inhibition of root growth was drastically reduced in LeMYC2 RNAi plants (Figure 6A-C). These results altogether indicate that LeMYC2 acts as a positive regulator of JA-mediated inhibition of root growth. We then monitored the expression of JA responsive genes, *LIN6* and *PIN2*, in LeMYC2 transgenic lines [43,56-58]. As shown in Figure 6D-E, JA treatment induced the expression of *LIN6* and *PIN2* genes in vector control plants, and the level of expression was significantly higher in LeMYC2 overexpresser transgenic plants. The level of induction of *LIN6* and *PIN2* expression was however drastically reduced in RNAi transgenic lines (Figure 6D-E). These results suggest that LeMYC2 promotes JA-induced expression of *LIN6* and *PIN2*.

## Discussion

The function of MYC2 has mainly been studied in Arabidopsis thus far [39,40,43]. In recent years, the functional homologs of MYC2 have been identified from other dicot and also monocot plants. Emerging evidences from other plant species suggest that MYC2 functions are broadly conserved in JA signaling pathways [43-45]. However, the possible role of MYC2 in plant growth and development remained unknown besides Arabidopsis [59]. This study reveals that the reduced level of LeMYC2 leads to hyperphotomorphogenic growth with shorter hypocotyl, whereas the higher level of LeMYC2 results in elongated hypocotyl as compared to wild type backgrounds. The expression of light regulated genes such as *LeRBCS-3A* and *LeCAB1* was also found to be up-regulated in RNAi and down-regulated in LeMYC2 overexpresser transgenic lines. Therefore, this study demonstrates that LeMYC2 works as a negative regulator of photomorphogenic growth and light regulated gene expression, and establishes the role of LeMYC2 in seedling development in tomato. It should be noted that although transgenic lines with similar level of LeMYC2 were chosen for this study, as shown in Figure 1, the level of LeMYC2 is not identical in the overexpresser lines. This slight alteration in the regulatory protein, LeMYC2, level in transgenic lines might have attributed to significant changes in the target gene expression.

The analyses of growth of transgenic seedlings overexpressing LeMYC2 or RNAi lines revealed that LeMYC2 predominantly works in BL-mediated seedling development. Altered level of chlorophyll and anthocyanin accumulation was also observed in the transgenic seedlings in BL. The function of LeMYC2, as observed from the phenotypic analysis of seedlings, remains confined to the lower fluences (5 μmol/m^2^/s) of WL without any significant change in chlorophyll accumulation. On the other hand, the overexpresser or RNAi transgenic lines of LeMYC2 grown at higher fluences (30 μmol/m^2^/s) of WL in the Green house exhibited strong phenotypic changes at the adult stage. It is worth mentioning here that although Arabidopsis *atmyc2* mutant seedlings do not exhibit morphological defects in WL, the adult plants display short stature with delayed flowering time in WL [39,52,60]. The adult transgenic tomato plants, including overexpresser and RNAi lines, in this study, did not display short stature. However, the LeMYC2 overexpresser transgenic plants grew with more branches with reduced internode distance, and the RNAi lines showed opposite phenotype. It has been shown earlier that overexpression of cry2 photoreceptor in tomato transgenic plants leads to dwarf phenotype with reduced internode distance [36]. Arabidopsis MYC2, the repressor of BL-mediated photomorphogenic growth, has been shown to work downstream to cry1 and cry2 photoreceptors [39]. In this study, it is observed that LeMYC2 overexpresser transgenic lines have increased number of branches with reduced internode distance. In addition to light, ABA and JA are also involved in plant growth and development [61,62]. Thus the plausible explanation of such observation could be attributed to the regulatory function of MYC2 in multiple signaling pathways including light, ABA and JA.

Although the fruit size remains the same, alteration in the seed size is observed in the LeMYC2 overexpresser and RNAi transgenic lines. Whereas the seed size is increased in the LeMYC2 overexpresser lines, it has been drastically reduced in the RNAi lines as compared to the control plants. Alteration in seed size has been reported with posttranslational silencing of *INVINH1*, an inhibitor of a cell wall invertase [63]. The microarray studies using Arabidopsis *atmyc2* mutants have shown that MYC2 controls the regulation of expression of genes involved in cell wall biosynthesis. LeMYC2 overexpression in tomato increases anthocyanin levels, which is reminiscent of the tomato high pigment mutants *hp1*and *hp2*, which display increased anthocyanin accumulation and shortened hypocotyl and internodes [23,24].

Negative regulators of photomorphogenesis such as COP1, SHW1 and MYC2 act as positive regulators of lateral root formation without any effect on root length [39,64,65]. This study shows that in addition to lateral root formation, LeMYC2 also promotes the root length of tomato plants. The light-regulated gene expression studies reveal that LeMYC2 represses the expression of *CAB* and *RBCS* genes. The light-mediated induction kinetics demonstrates that *LeRBCS-3A* expression is significantly higher in the dark grown seedlings in RANi transgenic lines. Thus, although LeMYC2 transgenic lines do not show any altered morphology in the dark, LeMYC2 plays a negative regulatory role in the expression of *LeRBCS-3A* in the darkness. The DNA-protein interaction studies reveal that LeMYC2 is able to bind to the G-box of *LeRBCS-3A* promoter. Interestingly, two protein-DNA complexes are detected that are equally completed out with excess unlabeled G-box, however not with the mutated version of the G-box. MYC2 in Arabidopsis acts as a transcriptional regulator in ABA and JA signaling pathways in Arabidopsis [40,42,43,66-71]. The ABA and JA responsiveness of LeMYC2 transgenic lines demonstrate that RNAi transgenic lines are less sensitive to ABA and JA mediated inhibition of seed germination and root growth, respectively. The effect of higher level of LeMYC2 was comparatively less than the reduced level (in RNAi lines) both in ABA or JA responsiveness.

## Conclusions

This study demonstrates the functions of *MYC2* in light, ABA and JA signaling pathways in tomato. Our data suggest that LeMYC2 is an important regulator of growth and development from seedling to flowering plants. The observations made in this work highlight strong biotechnological potential of LeMYC2 in crop improvement.

## Methods

### Plant material and growth condition

Tomato (*Lycopersiconesculentum*cv. Pusa ruby) seeds were obtained from the Indian Agricultural Research Institute, New Delhi. Seeds were sterilized, rinsed in sterile water, and sown in Magenta boxes containing full strength of Murashige and Skoog medium added with R3 vitamin (0.5 mg L–1 thiamine, 0.25 mg L–1 nicotinic acid, and 0.5 mg L–1 pyridoxine), 3% (w/v) Sucrose, and 0.8% (w/v) agar, pH 5.7. Tomato plants were grown initially in a growth room at 14-h-day and 10-h-night conditions at 24 to 25°C. Four-week-old seedlings were transferred to greenhouse conditions at natural day length (14 and 10 h light in the summer and winter, respectively) and standard condition (25°C day, 18°C night; 12 h watering cycle). Transgenic generation 1 (T1) populations of*LeMYC2*over expresser and RNAi plants were planted in the greenhouse. For hypocotyl measurements, T2 populations of transgenic and vector control lines were germinated in MS-agar in sterile jars under continuous white light, blue light or darkness at 22°C. Hypocotyl length measurement was performed with the help of Image J 1.41 software (NIH, USA)

### Plasmid construction and plant transformation

All transgenic constructs were made in plasmid of PBI121 binary vector, which in commonly used for *Agrobacterium tumefaciens*- mediated transformation of plant tissues [72,73]. The *LeMYC2*coding regions were amplified by PCR using synthetic primers *J2D*: 5′-GTGTTTATGGAATGAC-3′; *J2R*: 5′- GACGATTTCTATCTAC-3′ from 10 days old tomato seedlings. The full length tomato LeMYC2 was first cloned in pGEMT easy vector to generate compatible restriction site and was subsequently recloned in pBI121 by using Xba1 and BamH1 restriction site. To generate *LeMYC2*RNAi construct, the bHLH domain of 670 bp was removed from the *LeMYC2*/PBI121 construct using Xba I and BamHI and 430 bp from the start codon (ATG) of *LeMYC2*cDNA was amplified by PCR using forward primer T-RNAi (BamHI)FP: 5′ CGGGATCCATGACTGAATACAG 3′ (with BamHI as a flanking sequence) and reverse primer T-RNAi (XhoI)RP: 5′ CCGCTCGAGCCTTTTTGCTTTATC 3′ (with Xho1 as a flanking sequence). Amplified product was double digested with BamH1 and Xho1 and ligated in reverse orientation with previously digested LeMYC2/PBI121clone.

The pBI121 plasmid containing the *LeMYC2* full length or RNAi construct and only vector control were transformed into *A. tumefaciens* strain LBA4404 by the freeze-thaw method [74]. For Agrobacterium-mediated transformation and regeneration of tomato, cotyledons from 2-week-old seedlings were used as described previously [75,76]. Briefly, tomato seeds were sterilized using 4% Sodium Hypochlorite and germinated on Murashige and Skoog medium. After 2 weeks of germination the cotyledons were cut and co-cultivated 30 mins with the *A. tumefaciens* strain LBA4404 harboring the different constructs and kept in dark for 2 days. After 2 days of cocultivation the cotyledons were collected for selection on MS plates containing 50 mg/l kanamycin. When the plantlets regenerated, those were transferred to MS rooting medium [75,76]. Transgenic seeds were germinated in MS medium containing 150 μg/ml kanamycin to get the progeny plants.

The presence of the transgene in the regenerating plantlets was confirmed by PCR using the forward primer 5′ CGTTCCAACCACGTCTTCAAAGC 3′, which anneals to the *35S promoter* region, and the reverse primer 5′ CGAATATCTGCATCGGCGAACTG 3′ for over expresser lines, respectively, which anneal to the *LeMYC2*coding region. Genomic DNA was isolated using a commercial kit (DNeasy Plant Mini Kit; Qiagen,Valencia, CA, USA).

### Detection of small interference RNA (Si RNA) by northern blot

Total RNAwas extracted using Tripure reagent according to the protocol provided by the manufacturer (Roche, http://www.lifetechnologies.com/in/en/home.html). Total RNA (approximately 100 μg) was fractionated on 15% polyacrylamide/8 M urea denaturing acrylamide gel in 1X TBE buffer mirVaana TM miRNAisolatation kit (Applied Biosystem). Before loading total RNA was mixed with equal volume of gel loading bufferll (mirVana™miRNAisolatation kit and denaturated at 95°C for 5 minutes). Gel was electrophoresed at 30–45 mA till completion of the run (stopped electrophoresis when the bromophenol blue dye front had migrated to the bottom of gel). After that, the gel was soaked for 5 minutes in 0.5-1 ug/mL solution of ethidium bromide in 1X TBE and washed the gel for 2–5 mins in 1X TBE. Visulized the RNA using a UV transiluminator to make sure that there is good separation of RNA and photograph was taken for control. After staining, RNA was transferred to nylon membrane (pre equilibrated in 0.25X TBE), Hybond N (GE healthcare) by capillary transfer in 0.25X TBE for 16 h Nylon membrane was rinsed in 6X SSC, cross-linked in UV crosslinker at 254 nm for 1 minute 45 seconds and 1.5 J/cm^2^, dried and stained in 0.02% methylene blue in 0.3 M CH3COONa. Excess stain was washed with DEPC treated water. The membrane was pre-hybridized for 2–4 hrs at 65°C in prehybridisation buffer containing 6X SSC, 10X Denhard’s solution and 0.2% SDS. After prehybridization, radiolabelled probe (pre-denatured by boiling for 10 min and 68 snap-cooled for 3–5 min. at 4°C) in hybridization solution (6X SSC, 5X Denhard’s solution and 0.2% SDS) was added. After 16-18 h of incubation at room temperature in the hybridization solution, the membrane was washed twice with 2X SSC, 0.1% SDS at room temperature for 5 min and checked the count. If differential count has not been achieved membrane was subsequently washed twice with 0.2X SSC and 0.2% SDS at 42°C for 5–10 min and twice with 0.1X SSC and 0.1% SDS at 42°C for 5 min. After the final wash, the membrane was wrapped in plastic wrap and then exposed to X-ray film or a phosphorimager screen for autoradiography.

### Protein extraction and Western blot analysis

Total Protein Extraction - The seedlings (100 mg) were frozen in liquid nitrogen and ground in 200 μl of grinding buffer (400 mM sucrose, 50 mMTris-Cl pH 7.5, 10% glycerol, 2.5 mM EDTA) and PMSF was added (0.5 μl for every 100 μl of grinding buffer). The protein extract was transferred to fresh microcentrifuge tube and centrifuged at 10000 rpm for 10 min to pellet down the debris. The supernatant was transferred to fresh tube and an aliquot of 3 μl was taken out in a separate tube for the estimation of protein by Bradford assay. To the rest of the protein extract, appropriate volume of 5X sample buffer (200 mMTris- Cl pH 6.8, 400 mM DTT, 4% SDS, 0.025% Bromophenol blue, 20% glycerol) was added and boiled for 5 min before loading on SDS-PAGE. Western blot analysis - Western blot was performed using the Super signal west Pico chemiluminescent substrate kit (Pierce, USA) and following the instructions as described in users manual provided by the manufacturer.

### Quantitative real-time PCR

Control and transgenic seedlings were grown under required conditions. Total RNA was isolated using the RNeasy plant extraction kit (Qiagen) according to the manufacturer’s protocol. Two micrograms of total RNA was reverse transcribed to cDNA using a Titan One Tube RTPCR system (Roche Applied *Science)* following the manufacturer’s instructions. The quantitative real time PCR (qRT-PCR) was performed using One Step Real Time RT PCR (Applied Biosystems) with SYBR Green dye. The analysis was done in triplicate from cDNA derived from three independent experiments. Values were normalized with the amplification of endogenous reference gene (actin or tubulin) as a constitutively expressed internal control and relative to control. Primers used are as follows:

*LeMYC2* FP: 5′GCCACACTGGAGGCAAGATT3′ and RP: 5′ TTGCATCCCATCCGATGAT 3′

Tubulin-FP: 5′GGCGCTCATTGGACATTGA3′ and RP: 5′ CCTGTGAAATAAGGCGGTTAAGA 3′

*Le-CAB1*FP: 5′TGGTTCATGCACAAAGCATC3′ and RP: 5′ TCACTTT.CCGGGAACAAAGT 3′.

*Le-RBCS3A*FP: 5′CTTTGGGTTTTCCCTTGAGA3′ and RP: 5′ TTGGAGTCAATCGAGGGAGTA 3′

*ACTIN2* FP: 5′TGATGCACTTGTGTGTGACAA3′ and RP: 5′ GGGACTAAAACGCAAAACGA 3′

*LIN6* FP: 5′ACCCAAAAGGAGCAACATGGGG3′ and RP: 5′ CCATCAATAGAAGTGTTATCCGG 3′

*PIN2* FP: 5′CCCACGTTCAGAAGGAAGTC3′ and RP: 5′ TTTTGGGCAATCCAGAAGAT 3′

*AREB* FP: 5′GCACTCAACTCTAATTTCATTCAAGG3′ and RP: 5′TACGTATTTCCTGCCTCTTAAACC 3′

*PP2C* FP: TCGGAAGGAGAAGATTACG3′ and RP: 5′ TCCACAATTCGCAACAAC 3′

*TAS14* FP: 5′ACAATACGGCAATCAAGACCAAATG3′ and RP: 5′ CCCATCATACCGCCAGTACCC 3′

### Chlorophyll and anthocyanin estimation

Leaf Tissue was ground in liquid N2. Total chlorophyll was extracted into 80% acetone, and chlorophyll a and b content was calculated by using MacKinney’s coefficients, in which chlorophyll a equals 12.7(*A*663) - 2.69(*A*645) and chlorophyll b equals 22.9(*A*645) -4.48(*A*663). For anthocyanin measurement leaf tissue (100-150 mg) or 3–4 seedlings were extracted overnight in 500 μl of extraction solution (1% HCL in Methanol). Next day, seedlings were crushed after addition of 400 μl of sterile water. Finally chlorophyll was removed by adding 1 ml of chloroform and debris was removed by centrifugation and supernatant was collected into a fresh microcentrifuge tube. Then Spectrophotometric estimation was carried out by taking readings at the wavelengths of 530 nm and 657 nm. The total Anthocyanin content was calculated with the help of the following formula: (A_530_-0.33A_657_)/g of fresh weight.

### Lateral root growth

Seeds were on MS medium on magenta boxes and stratified at 4°C in dark conditions for 4 d to induce uniform germination. The boxes were kept in racks, and the seedlings were grown under constant white light conditions (90 *μ*mol m^-2^ s^-1^) for 14 days. The root length and number of lateral roots of control and different LeMYC2 transgenic lines were measured. Approximately 15 seedlings were used for the root length and lateral root measurement.

### ABA and JA responsiveness

The seeds of vector control, LeMYC2overexpresser and LeMYC2 RNAitransgenic lines were sterilized with 4% sodium hypochlorite solution for 12 min and washed with sterile water for five times. The seeds were plated on to MS plates with 5 μM concentration of ABA (Sigma-Aldrich). The plates were kept in cold in dark for 4 days for stratification and then transferred to constant white light. The seeds were monitored for germination and greening from 3 days to 8 days. The seeds were counted as germinated when the radicle tip had fully expanded the seed coat. Control and different transgenic lines were germinated on MS with 10 μM of methyl jasmonate (Sigma) in magenta boxes, after stratification boxes were transferred to growth room and the root growth was monitored regularly and seedling were taken on plates and photographed and root length and lateral roots were measured.

## Competing interests

In the past five years the authors received no reimbursements, fees, funding, or salary from National Institute of Plant Genome Research (NIPGR), New Delhi, India that may in any way gain or lose financially from the publication of this manuscript. The article processing fees may be paid by NIPGR, New Delhi, India. We do not hold any stocks or shares of the organization. Since part of this work has already been patented (References: 51), we/NIPGR may sell the patents in future.

## Authors’ contributions

NG was involved in generation of the RNAi transgenic lines, phenotypic characterization, JA, ABA responsiveness and gene expression study. VBRP cloned LeMYC2 and generated the overexpresser transgenic lines and phenotypic analyses. SC conceived of the study, participated in its design and coordination, and helped to draft the manuscript. All authors read and approved the final manuscript.

## Supplementary Material

Additional file 1: Figure S1 The LeMYC2 transcription factor shows strong similarity with other homologues sequences. A, Comparison of amino acid sequence of LeMYC2 transcription factor with other homologues sequences from Arabidopsis (AtMYC2, AtbHLH06) *Catharanthus roseus* CrMYC2 (AAQ14332), and *Solanum tuberosum* (JAMYC2, CAF74710). B, Unrooted phylogenetic tree of the deduced amino acid sequences of LeMYC2 and other plant MYC proteins. The phylogenetic tree was generated based on an alignment of the full length deduced amino acid sequences of 24 MYC proteins, including, *Vitisvinifera*VvMYC2 (ABR23669); *Arabidopsis thaliana* AtMYC1 (AtbHLH12; D83511), AtMYC2 (AtbHLH06; Q39204), AtMYC3 (AtbHLH121; Q9FIP9) and AtMYC4 (AtbHLH080; 049687); *Catharanthus roseus*CrMYC1 (BAF42667), CrMYC2 (AAQ14332), CrMYC3 (FJ004233), CrMYC4 (FJ004234) and CrMYC5 (FJ004235); *Taxus cuspidate*TcJAMYC2 (ACM48567); *Nicotiana tabacum* NtMYC1a (ADH04267) , NtMYC1b (ADH04268) ,NtMYC2a (ADU60100) and NtMYC2b ( ADU60101); *Brassica napus* BnMYC2 (CCQ71910); *Hevea brasiliensis* HbMYC2 (ACF19982); *Cucumis sativus* CsMYC2 (XP_004148475), *Medicago truncatula* MtMYC2 (XP_003628820); *Glycine max* GmMYC2 (XP_003531962); *Zea mays* ZmMYC7E (AAD15818) *Solanum tuberosum* JAMYC2 (CAF74710), JAMYC10 (CAF74711) and LeJA3 (AAF04917). Alignments were made using CLUSTAL Omega multiple sequence alignment tool. The phylogenetic tree was constructed by the Maximum-likelihood approach using the MEGA5.10 program with default settings. LeMYC2 is shown in the bracket. Numbers at the branch points indicated bootstrap values based on 1000 bootstrap replicates. Click here for file
