# Supplementary figures and images for: LeMYC2 acts as a negative regulator of blue light mediated photomorphogenic growth, and promotes the growth of adult tomato plants
Source: BMC Plant Biol. 2014 Jan 31;14:38. doi: 10.1186/1471-2229-14-38 (PMC3922655; doi:10.1186/1471-2229-14-38)

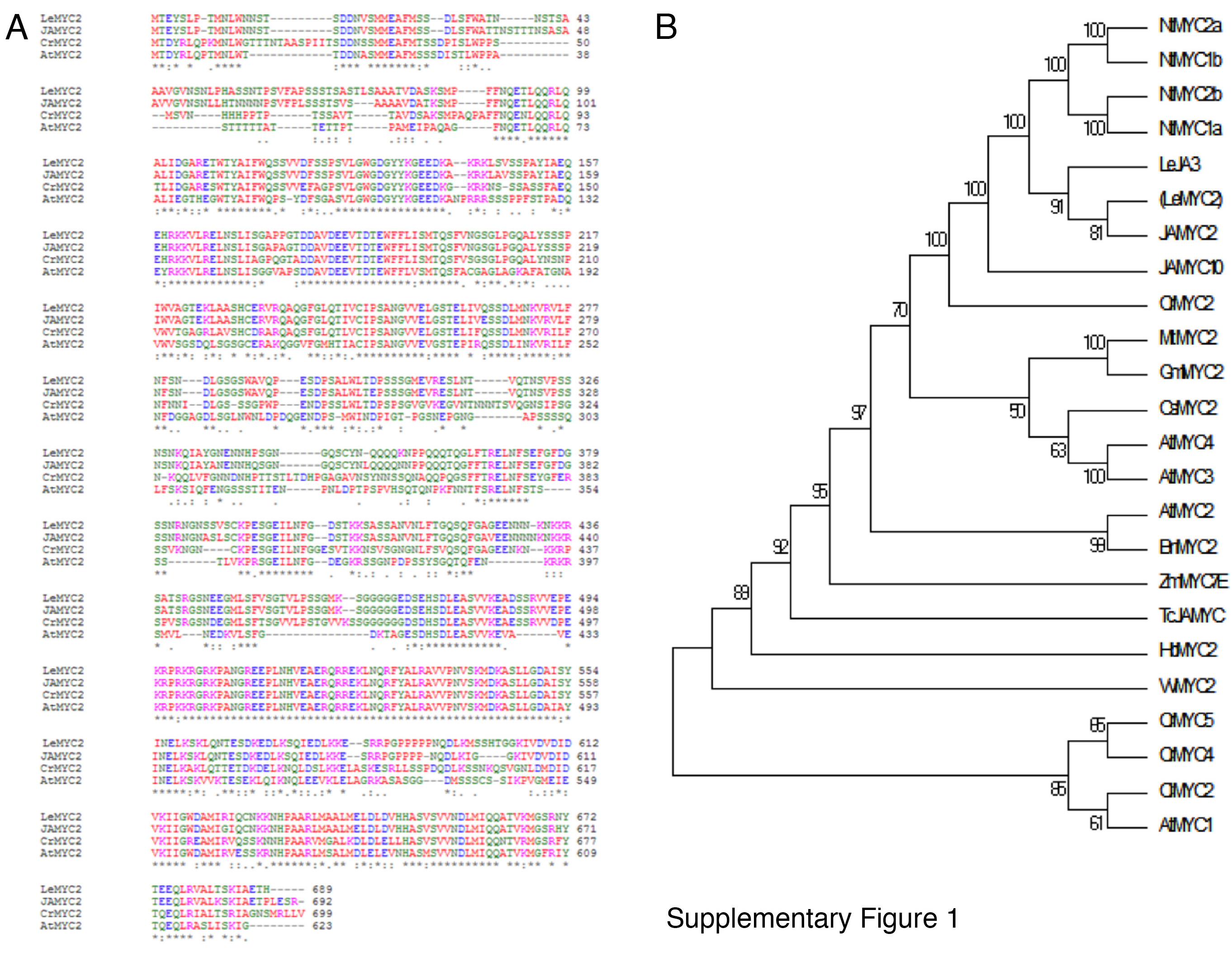

Supplement: Additional file 1: Figure S1 — The LeMYC2 transcription factor shows strong similarity with other homologues sequences. A, Comparison of amino acid sequence of LeMYC2 transcription factor with other homologues sequences from Arabidopsis (AtMYC2, AtbHLH06) Catharanthus roseus CrMYC2 (AAQ14332), and Solanum tuberosum (JAMYC2, CAF74710). B, Unrooted phylogenetic tree of the deduced amino acid sequences of LeMYC2 and other plant MYC proteins. The phylogenetic tree was generated based on an alignment of the full length deduced amino acid sequences of 24 MYC proteins, including, VitisviniferaVvMYC2 (ABR23669); Arabidopsis thaliana AtMYC1 (AtbHLH12; D83511), AtMYC2 (AtbHLH06; Q39204), AtMYC3 (AtbHLH121; Q9FIP9) and AtMYC4 (AtbHLH080; 049687); Catharanthus roseusCrMYC1 (BAF42667), CrMYC2 (AAQ14332), CrMYC3 (FJ004233), CrMYC4 (FJ004234) and CrMYC5 (FJ004235); Taxus cuspidateTcJAMYC2 (ACM48567); Nicotiana tabacum NtMYC1a (ADH04267) , NtMYC1b (ADH04268) ,NtMYC2a (ADU60100) and NtMYC2b ( ADU60101); Brassica napus BnMYC2 (CCQ71910); Hevea brasiliensis HbMYC2 (ACF19982); Cucumis sativus CsMYC2 (XP_004148475), Medicago truncatula MtMYC2 (XP_003628820); Glycine max GmMYC2 (XP_003531962); Zea mays ZmMYC7E (AAD15818) Solanum tuberosum JAMYC2 (CAF74710), JAMYC10 (CAF74711) and LeJA3 (AAF04917). Alignments were made using CLUSTAL Omega multiple sequence alignment tool. The phylogenetic tree was constructed by the Maximum-likelihood approach using the MEGA5.10 program with default settings. LeMYC2 is shown in the bracket. Numbers at the branch points indicated bootstrap values based on 1000 bootstrap replicates. [file 1471-2229-14-38-S1.jpeg]
